# Structural Distortion of Cycloalkynes Influences Cycloaddition Rates both by Strain and Interaction Energies

**DOI:** 10.1002/chem.201900295

**Published:** 2019-03-27

**Authors:** Trevor A. Hamlin, Brian J. Levandowski, Ayush K. Narsaria, Kendall N. Houk, F. Matthias Bickelhaupt

**Affiliations:** ^1^ Department of Theoretical Chemistry Amsterdam Center for Multiscale Modeling (ACMM) Vrije Universiteit Amsterdam De Boelelaan 1083 1081 HV Amsterdam The Netherlands; ^2^ Department of Chemistry and Biochemistry University of California Los Angeles CA 90095 USA; ^3^ Institute for Molecules and Materials (IMM) Radboud University of Nijmegen Heyendaalseweg 135 6525 AJ Nijmegen The Netherlands

**Keywords:** alkynes, azides, cycloaddition, density functional calculations, reaction mechanisms

## Abstract

The reactivities of 2‐butyne, cycloheptyne, cyclooctyne, and cyclononyne in the 1,3‐dipolar cycloaddition reaction with methyl azide were evaluated through DFT calculations at the M06‐2X/6‐311++G(d)//M06‐2X/6‐31+G(d) level of theory. Computed activation free energies for the cycloadditions of cycloalkynes are 16.5–22.0 kcal mol^−1^ lower in energy than that of the acyclic 2‐butyne. The strained or predistorted nature of cycloalkynes is often solely used to rationalize this significant rate enhancement. Our distortion/interaction–activation strain analysis has been revealed that the degree of geometrical predistortion of the cycloalkyne ground‐state geometries acts to enhance reactivity compared with that of acyclic alkynes through three distinct mechanisms, not only due to (i) a reduced strain or distortion energy, but also to (ii) a smaller HOMO–LUMO gap, and (iii) an enhanced orbital overlap, which both contribute to more stabilizing orbital interactions.

## Introduction

**Scheme 1:**
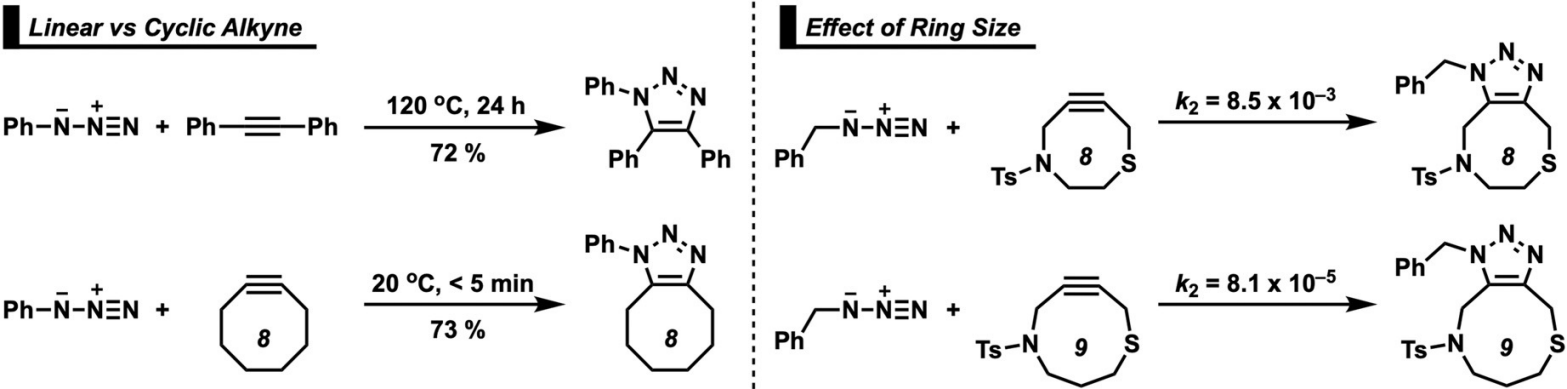
Cycloadditions of azides with acyclic^12^ and cyclic alkynes.^11^, ^13^ Ts=tosyl.

The Huisgen 1,3‐dipolar cycloaddition reaction of azides was originally uncatalyzed, often required high temperatures or pressures, and produced a mixture of products.^1^ The groups of Sharpless^2^ and Meldal^3^ reported copper‐catalyzed reactions of azides with aliphatic alkynes that rapidly “clicked” together to form 1,2,3‐triazoles in a regioselective and high‐yielding fashion under mild conditions. These robust copper‐catalyzed reactions have found a wide range of applications, including organic synthesis,^4^ drug discovery,^5^ chemical biology,^6^ and materials chemistry.^7^ The toxicity of copper, however, limits the scope of biological applications.^8^ Blomquist and Liu first reported the “strain‐promoted” reaction between cyclooctyne (**8yne**) and phenyl azide and remarked on the “explosive” nature of this transformation.^9^ The accelerated reactivity of cyclic alkynes, relative to acyclic alkynes, has been attributed to strain, and these reactions have been accordingly called strain‐promoted azide–alkyne cycloadditions (SPAACs; Scheme 1, left).^10^ The ring size of the cycloalkyne additionally affects the reaction rate. Scheme 1 (right) shows how a thiacyclooctyne is two orders of magnitude more reactive than a thiacyclononyne.^11^

Bioorthogonal reactions enable the labeling of biomolecules that were previously not traceable by green fluorescent protein studies.^14^ These reactions proceed rapidly and selectively in living systems without disrupting biological processes.^15^ The rate constants for copper‐free click reactions of strained alkynes with azides are fast enough for the in vitro and in vivo labeling of biomolecules.^16^ It has been pointed out that SPAAC reactions used in bioorthogonal chemistry generally result from predistortion of the reactants toward their transition‐state geometries.^17^ The use of SPAAC reactions in bioorthogonal chemistry has sparked interest in the relationship between strain and reactivity. To enhance the reactivity of the SPAAC, researchers have typically focused on the synthesis of highly strained cyclic alkynes, such as bicyclo[6.1.0]non‐4‐yne (BCN),^18^ azadibenzocyclooctyne (DIBAC),^19^ 4‐dibenzocyclooctynol (DIBO),6a difluorobenzocyclooctyne (DIFBO),^20^ and 3,3,6,6‐tetramethylthiacycloheptyne (TMTH).^21^ Although improving upon the reaction rates, the inherent strain, or distortion, also decreases the stability of the cycloalkyne and results in unwanted side reactions with biological nucleophiles. Alabugin and co‐workers developed strategies for enhancing the reactivity of the SPAAC.^22^ Their approach utilizes stereoelectronic effects that stabilize the transition state without compromising the stability of the cycloalkane. Substitution of the propargylic position with a heteroatom (N, O, or S)^11^, ^23^ or introduction of propargylic fluorides^24^ electronically activates the cyclic alkynes, leading to lower activation barriers through π*–σ*_C−X_ hyperconjugative interactions, which stabilize the transition state.

Our groups have previously discussed the role of predistortion on strain‐promoted cycloadditions and noted that cycloalkynes and cycloalkenes require less distortion to achieve the transition‐state geometry and react faster than that of acyclic dienophiles.^17^, ^25^ We recently discovered that secondary orbital interactions, in addition to distortion energy,^26^ had a significant influence on the Diels–Alder reactivity of strained cycloalkenes.^27^ In light of these findings and the fact that predistortion can act to both reduce the strain, or distortion energy, and enhance interactions, we have reinvestigated the reactivities of a model cycloalkyne series, cycloheptyne (**7yne**), cyclooctyne (**8yne**), and cyclononyne (**9yne**), and the acyclic alkyne 2‐butyne (**2yn**), with methyl azide (**Az**) by using the distortion/interaction–activation strain model.^17^, ^28^

## Computational Details

Geometry optimizations and vibrational frequency calculations were performed by using the M06‐2X^29^ density functional with the 6‐31+G(d) basis set in the Gaussian 09 (Revision D.01) suite.^30^ Single‐point energies were calculated at the M06‐2X/6‐311++G(d) level of theory. The M06‐2X functional was previously shown to provide relatively accurate energies for a number of cycloaddition reactions.^31^ Normal mode analysis was used to verify each stationary point as either a minimum or a first‐order saddle point. The thermal corrections were computed from unscaled M06‐2X/6‐31+G(d) frequencies for a 1 m standard state and 298.15 K.

The 1,3‐dipolar cycloadditions reactions were analyzed by using the distortion/interaction–activation strain method (D/I‐ASM)^28^, ^32^ to elucidate the factors that gave rise to the enhanced reactivity of cycloalkynes. This analysis was performed by using the ADF.2016.102 program^33^ at the M06‐2X/TZ2P^29^, ^34^ level of theory on the M06‐2X/6‐31+G(d) geometries optimized in Gaussian 09.^30^ The D/I‐ASM analysis decomposed the electronic energies, Δ*E*(*ζ*), into two terms: the strain, or distortion energy, Δ*E*_strain_(*ζ*), associated with distorting the individual reactants and the interaction, Δ*E*_int_(*ζ*), between the deformed reactants along the intrinsic reaction coordinate (IRC), which was projected upon the average distance of the two newly forming C⋅⋅⋅N bonds [Eq. (1)].

(1) $$\Delta E\left(\zeta \right)=\Delta E{}_{\mathrm{strain}}\left(\zeta \right)+\Delta E{}_{\mathrm{int}}\left(\zeta \right)$$

The latter reaction coordinate, *ζ*, was critically involved in the essential chemical transformation of forming new bonds and underwent a well‐defined change over the course of the reaction.^35^ The interaction term was further assessed by a canonical energy decomposition analysis (EDA), which decomposed the Δ*E*_int_(*ζ*) term into three physically meaningful terms: 1) Δ*V*_elstat_(*ζ*), the classical electrostatic interactions; 2) Δ*E*_Pauli_(*ζ*), closed‐shell repulsions (steric effects); and 3) Δ*E*_oi_(*ζ*), charge transfer, including HOMO–LUMO interactions, and polarization [Eq. (2)].

(2) $$\Delta E{}_{\mathrm{int}}\left(\zeta \right)=\Delta V{}_{\mathrm{elstat}}\left(\zeta \right)+\Delta E{}_{\mathrm{Pauli}}\left(\zeta \right)+\Delta E{}_{\mathrm{oi}}\left(\zeta \right)$$

The strain, or distortion energy, and interaction energy terms were highly dependent on where the transition state was located on the reaction coordinate.^36^ Thus, to guarantee a consistent comparison, we analyzed the geometries of the cycloaddition reactions at constant average C⋅⋅⋅N bond‐forming length (2.22 Å). Importantly, these consistent geometries had energies very close to, or exactly the same as, that of the transition‐state energies. The trend in 2.22 Å geometry energies mirrored the trend in activation energies.

## Results and Discussion

**Figure 1:**
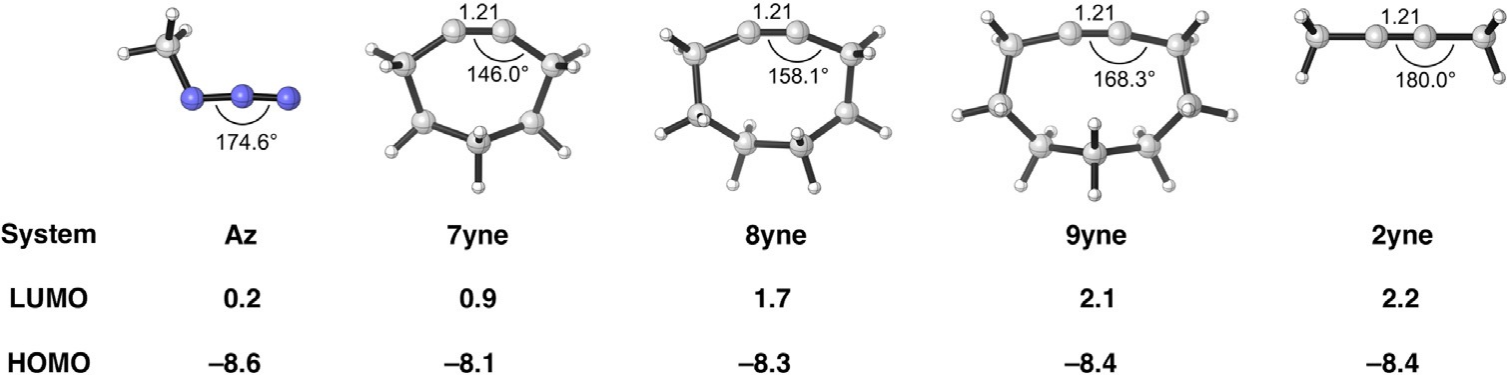
C≡C bond lengths [Å], internal angles [°] [computed at M06‐2X/6‐31+G(d)], and HOMO and LUMO [eV] [computed at M06‐2X/TZ2P//M06‐2X/6‐31+G(d)] of the 1,3‐dipole and alkynes included in the present study calculated.

The optimized geometries of the ground‐state reactants are shown in Figure 1. The cycloalkynes, **7yne**–**9yne**, and acyclic alkyne, **2yne**, have the same C≡C bond lengths of 1.21 Å. The internal C‐C‐C bond angle of the alkyne is highly dependent on ring size. The alkyne bending angle systematically increases as the ring becomes larger along the series **7yne**–**9yne**, at 146.0, 158.1, and 168.3°, respectively. Without the influence of ring strain, the acyclic **2yne** is linear.

**Figure 2:**
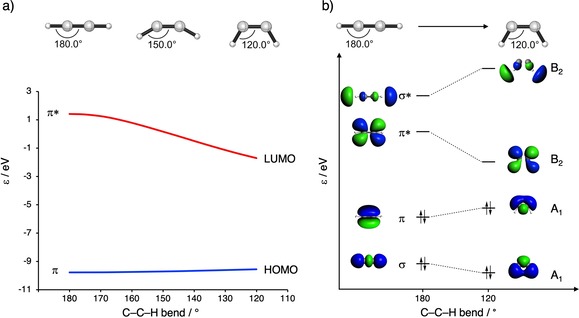
a) FMO energies [eV] associated with the bending of acetylene from 180 to 120°, calculated at M06‐2X/TZ2P, and b) schematic Walsh diagram explaining the effect of bending in acetylene on orbital energies.

One of our groups noted that the bending of alkenes and alkynes had a profound effect on the energy of the frontier molecular orbitals (FMO).^37^ The calculated HOMO and LUMO energies of the strained alkynes, **7yne**–**9yne**, and the unstrained alkyne, **2yne**, are shown in Figure 1. The LUMO energies of the alkynes range from 0.9 to 2.2 eV, whereas the HOMO energies change to a lesser extent, from −8.1 to −8.4 eV. Figure 2 a shows how in‐plane bending alters the HOMO and LUMO levels in acetylene. Bending of the C−H bonds leads to overlap of σ‐C−H with the π bonds. This increased overlap results in a stabilization of the LUMO and a slight destabilization of the HOMO.^37^ Figure 2 b shows that the LUMO is stabilized upon bending by the in‐phase mixing of the σ* and π* orbitals. Conversely, the HOMO is destabilized by the out‐of‐phase mixing of the acetylene σ and π orbitals upon bending. This mixing is primarily due to overlap between the H 1 s lobes of the σ and σ* molecular orbitals (MOs) with the C 2 p_π_ lobes of the π and π* MOs, respectively. Thus, orbital mixing between the σ and π MOs of bent acetylene raises the HOMO and lowers the LUMO energy. The effect is much larger on the LUMO than that of the HOMO because introduction of the 2 s character stabilizes both orbitals.^37^

**Figure 3:**
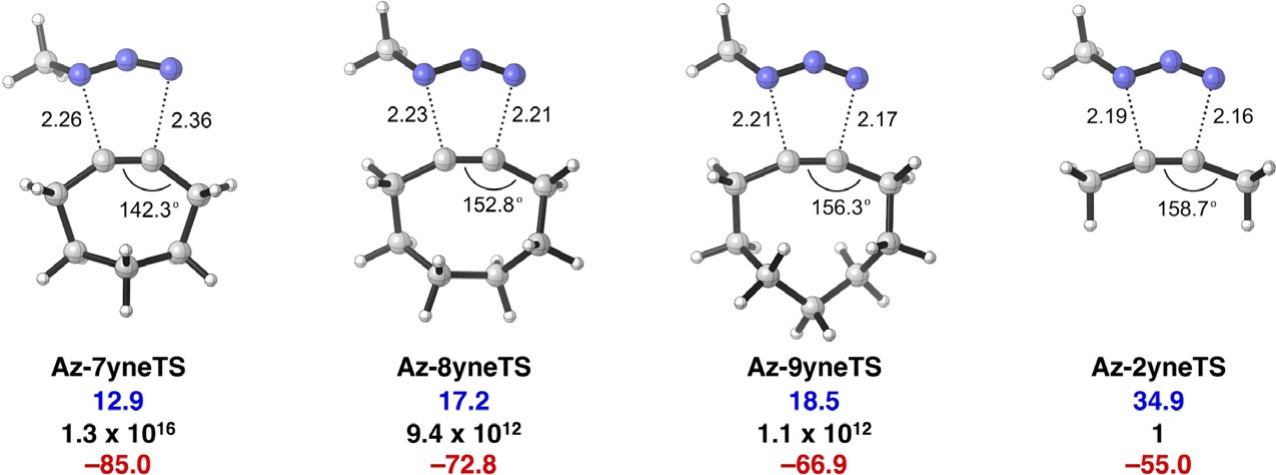
Transition structures with forming bond lengths (in Å), computed Gibbs activation free energies (Δ*G* ^≠^, blue, in kcal mol^−1^), relative rate constants (*k*_rel_, black), and Gibbs reaction free energies (Δ*G*_rxn_, red, in kcal mol^−1^), for the cycloaddition reactions of **2yne**, **7yne**, **8yne**, and **9yne** with **Az**, computed at M06‐2X/6‐311++G(d)//M06‐2X/6‐31+G(d).

Figure 3 shows the transition states for the 1,3‐dipolar cycloadditions of **Az** with the cycloalkynes (**Az‐7yneTS**–**Az‐9yneTS**) and 2‐butyne (**Az‐2yneTS**). Transition structures become earlier, with regard to their newly forming bonds, as the ring size of the cycloalkyne decreases and are most product‐like in the case of unstrained **2yne**. The Gibbs activation free energies (Δ*G* ^≠^) and reaction energies (Δ*G*_rxn_) are presented below each structure. The cycloaddition of **7yne** is predicted to proceed rapidly, with a low activation barrier (12.9 kcal mol^−1^), via an early transition structure and is highly exergonic (−85.0 kcal mol^−1^). The cycloadditions for **8yne** and **9yne** have higher barriers of 17.2 and 18.5 kcal mol^−1^, respectively, and are less exergonic compared with **7yne**. The cycloaddition of the acyclic alkyne, **2yne**, with **Az** has a very high barrier (Δ*G* ^≠^=34.9 kcal mol^−1^) and is less exergonic, relative to the cycloalkyne cycloadditions. The predicted relative rates (*k*_rel_) span 16 orders of magnitude across the cycloadditions (see Figure 3).

**Figure 4:**
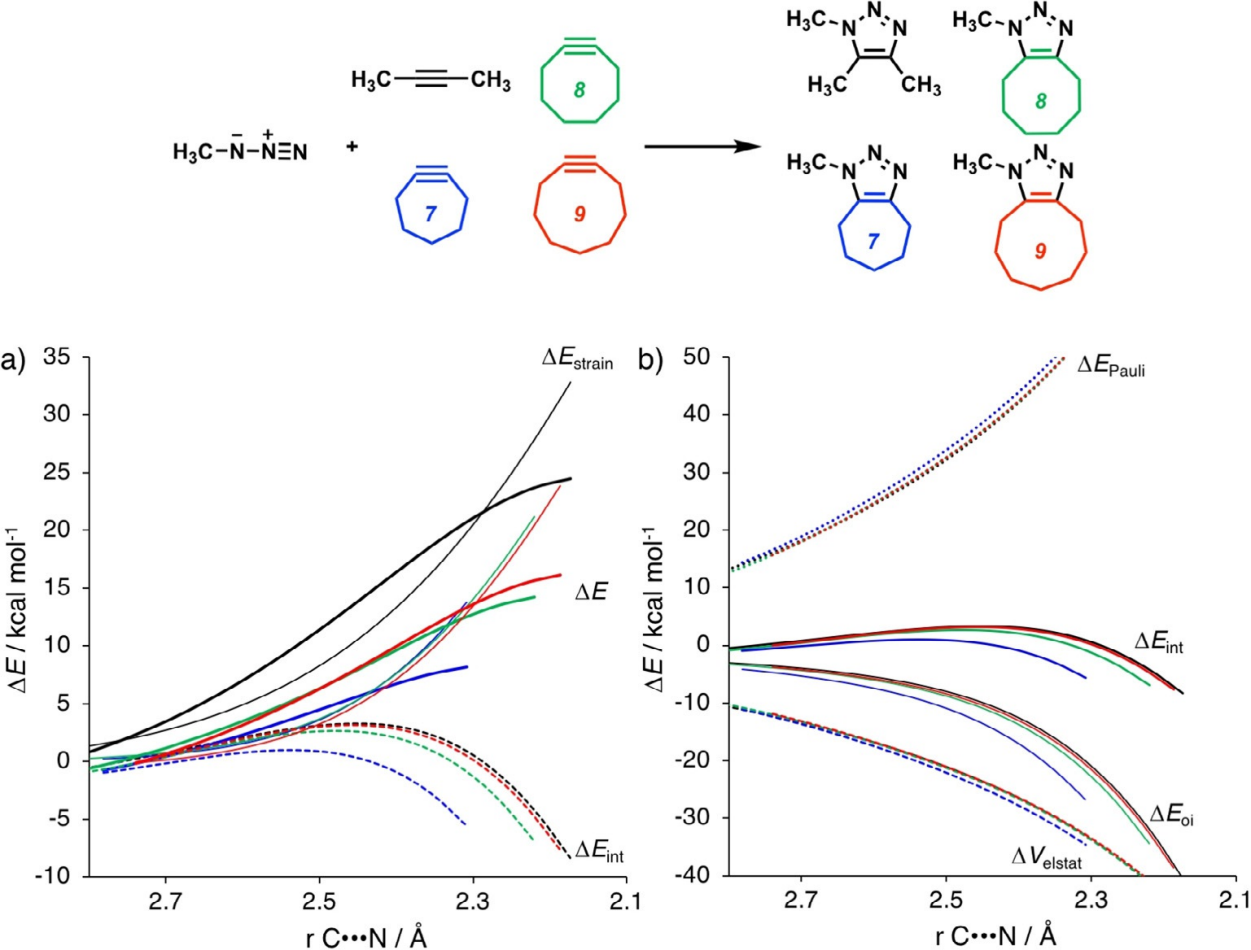
a) Distortion/interaction–activation strain analyses and b) EDAs of the cycloaddition reactions of **Az** with alkynes (black, **2yne**; blue, **7yne**; green, **8yne**; red, **9yne**). All data were computed at M06‐2X/TZ2P//M06‐2X/6‐31+G(d).

Figure 4 graphically represents how the computed strain, or distortion energy, (Δ*E*_strain_) and interaction (Δ*E*_int_) energy components evolve along the reaction coordinate for the 1,3‐dipolar cycloaddition between **Az** and the alkynes, **2yne** and **7yne**–**9yne**. Figure 4 a reveals that the accelerated reactivity of the cycloalkynes (**7yne**–**9yne**) relative to **2yne** results from a decrease in strain, or distortion energy, Δ*E*_strain_, along the reaction coordinate. Cycloalkynes are predistorted towards the transition‐state geometry and less bending of cycloalkynes is required during bond formation. Because the strain curves of the cycloalkynes are very similar, the origin of the increase in reactivity as the ring size of the cycloalkynes decreases can be attributed to differences in Δ*E*_int_.

Provided in Table 1 are the distortion/interaction–activation strain terms (Δ*E*, Δ*E*_strain_, and Δ*E*_int_) along with the EDA terms (Δ*E*_Pauli_, Δ*V*_elstat_, Δ*E*_oi_) calculated at a consistent geometry (2.22 Å). The values of Δ*E*_strain_ for the reactions involving **7yne**–**9yne** are similar and range from 20.5 to 21.6 kcal mol^−1^, whereas **2yne** has a much more destabilizing Δ*E*_strain_ of 28.4 kcal mol^−1^. The cyclic alkynes are much more reactive due to the decrease in Δ*E*_strain_ relative to the acyclic case. Interestingly, the decrease in Δ*E*, and thus, enhanced reactivity observed upon moving from **9yne** to **7yne** is paralleled by an increase in the strength of Δ*E*_int_. Thus, reactivity differences for the cycloalkynes in the 1,3‐dipolar cycloaddition with **Az** originate from differences in Δ*E*_int_ and not Δ*E*_strain_.

**Table 1:** Distortion/interaction–activation strain and EDA terms computed at consistent geometries with average C⋅⋅⋅N bond‐forming distances of 2.22 Å, for the cycloaddition reactions.^[a]^

Compound	Δ*E*	Δ*E*_strain_	Δ*E*_int_	Δ*E*_Pauli_	Δ*V*_elstat_	Δ*E*_oi_
**7yne**	8.2	21.6	−13.3	70.0	−42.7	−40.6
**8yne**	14.2	21.2	−6.9	68.7	−41.2	−34.4
**9yne**	15.6	20.5	−4.9	68.9	−41.0	−32.8
**2yne**	23.8	28.4	−4.6	70.0	−41.8	−32.9

[a] Δ*E*_int_(*ζ*)=Δ*V*_elstat_(*ζ*)+Δ*E*_Pauli_(*ζ*)+Δ*E*_oi_(*ζ*), see the Computational Details section for details. All data (in kcal mol^−1^) were computed at M06‐2X/TZ2P//M06‐2X/6‐31+G(d).

Table 2 summarizes the extent to which the cycloalkyne reaction is promoted by the differences in strain and interaction energies, relative to the reaction of the acyclic alkyne. Predistortion reduces the strain and promotes the reactivity of the cycloalkynes by five to six orders of magnitude. The more stabilizing interaction imparted by the predistorted geometry additionally enhances the reactivity of **8yne** by an order of magnitude and **7yne** by six orders of magnitude. In total, predistortion promotes the reactivity of the cycloalkynes by six to eleven orders of magnitude compared to 2yne and enhanced interactions begin to play an increasingly important role in the accelerated reactivity as the size of the cycloalkyne decreases.

**Table 2:** Relative rates (*k*_rel_) from electronic energies and the contribution of the strain and interaction energies to the differences in these relative rates.^[a]^

Compound	Predistortion promotion *k*_rel_^[b]^	Strain promotion *k*_rel,strain_^[b]^	Interaction promotion *k*_rel,int_^[b]^
**2yne**	1.0	1.0	1.0
**9yne**	1.0×10^6^	6.2×10^5^	1.7
**8yne**	1.1×10^7^	1.9×10^5^	4.9×10^1^
**7yne**	2.7×10^11^	9.7×10^4^	2.4×10^6^

[a] All data were computed at M06‐2X/TZ2P//M06‐2X/6‐31+G(d) at consistent geometries with average C⋅⋅⋅N bond‐forming distances of 2.22 Å. [b] Analysis of relative rates: *k*_rel_=*k*_***X*****yne**_/*k*_**2yne**_=*e* ^−[Δ*E*(***X*****yne**)−Δ*E*(**2yne**)]/RT^=*e* ^−ΔΔ*E*/*RT*^=*e* ^−ΔΔ*E*strain/*RT*^ *e* ^−ΔΔ*E*int/*RT*^=*e* ^−ΔΔ*E*/*RT*^=*k*_rel,strain_ *k*_rel,int_.

**Figure 5:**
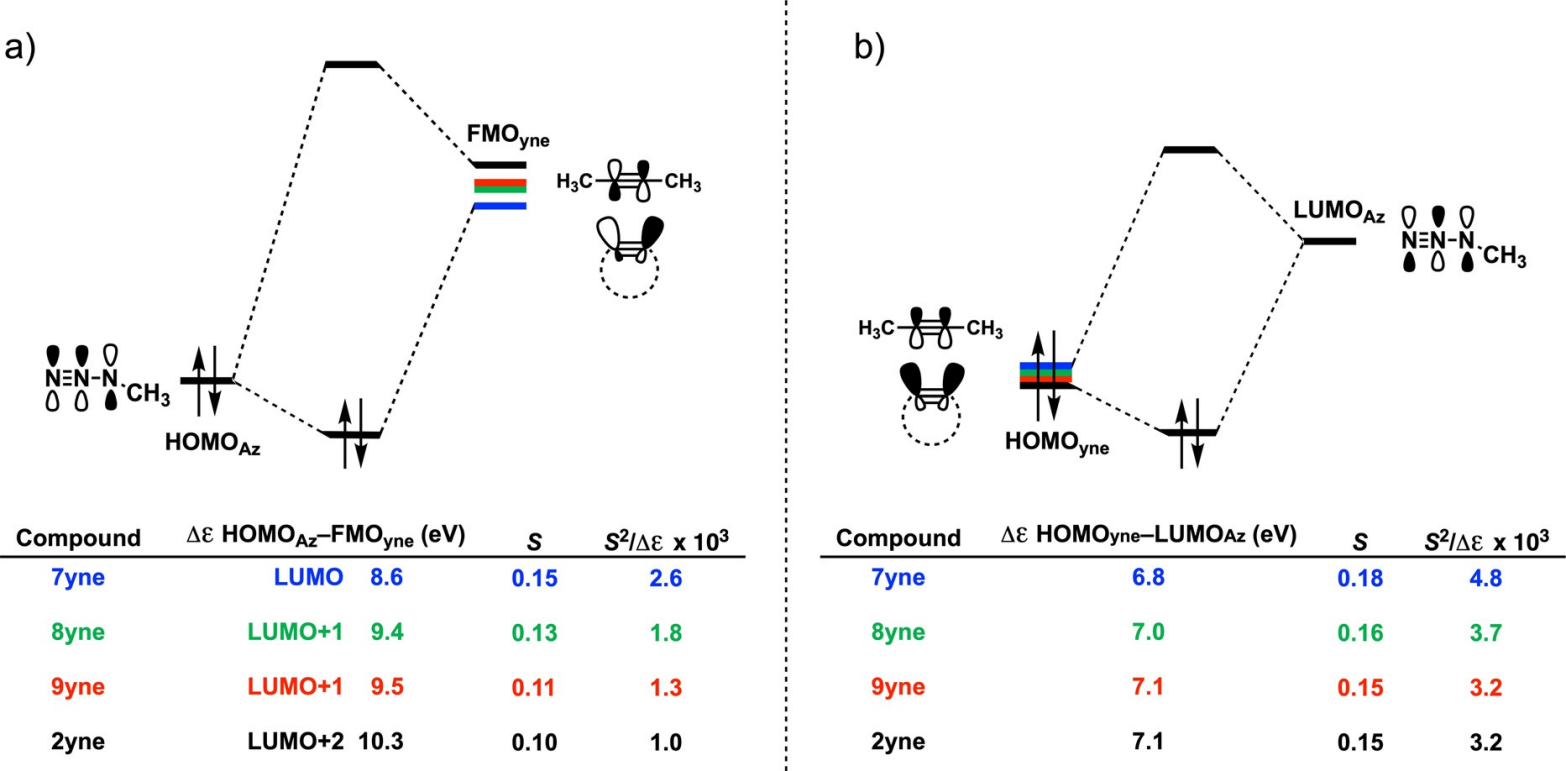
MO diagram, with the key orbital energy gap, overlap, and the *S* ^2^/Δ*ϵ* terms of a) the HOMO_**Az**_–FMO_**yne**_ interaction and b) the HOMO_**yne**_–LUMO_**Az**_ interaction for cycloaddition reactions between **Az** and alkynes **2yne** and **7yne**–**9yne**. All data were computed at M06‐2X/TZ2P//M06‐2X/6‐31+G(d).

The components of total Δ*E*_int_ have been analyzed by means of the EDA method, and the results are shown in Figure 4 b and are summarized in Table 1. Differences in the curves of Δ*E*_Pauli_ and Δ*V*_elstat_ are minimal, as are their absolute values at a consistent geometry. However, differences in the strengths of the orbital interactions, Δ*E*_oi_, are more pronounced and responsible for determining the trends in Δ*E*_int_. The value of Δ*E*_oi_ is most stabilizing for **7yne** (−40.6 kcal mol^−1^) and diminishes as the ring size increases to **9yne** (−32.8 kcal mol^−1^). Importantly, differences in the reactivity of cycloalkynes are the result of differences in Δ*E*_oi_ and not Δ*E*_strain_. To further probe the key orbital interactions involved in the 1,3‐dipolar cycloadditions of **2yne** and **7yne**–**9yne** with **Az**, we analyzed the MOs participating in these interactions (Figure 5). MO diagrams and orbital overlaps were calculated at the M06‐2X/TZ2P//M06‐2X/6‐31+G(d) level by using Kohn–Sham MO analyses.^38^ These orbital analyses were carried out on consistent geometries with average C⋅⋅⋅N bond‐forming distances of 2.22 Å.

**Figure 6:**
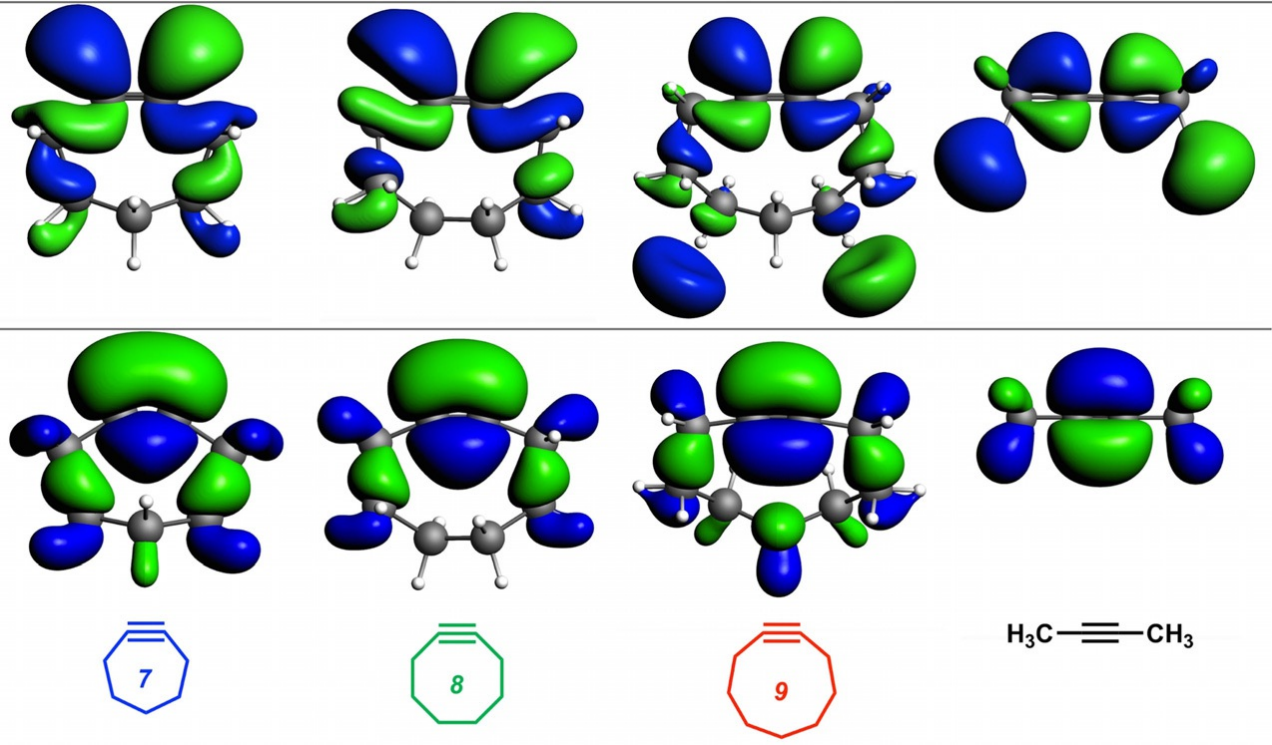
FMO diagram (isovalue=0.03) for ground‐state reactants **2yne**, **7yne**, **8yne**, and **9yne** (top row: interacting virtual orbitals, bottom row: interacting occupied orbitals). The two rows of molecules are aligned horizontally along the C≡C bond and solid lines are positioned along the top of the FMO of **7yne** to illustrate the extent of π‐ and π*‐orbital hybridization as a function of ring strain. All data were computed at M06‐2X/TZ2P//M06‐2X/6‐31+G(d).

The normal electron demand (NED) interaction for the cycloaddition reactions between **2yne** or **7yne**–**9yne** and **Az** occurs between the HOMO_**Az**_ and unoccupied FMO_**yne**_ (Figure 5 a). Here, FMO_**yne**_ refers to the virtual π‐FMO of the (cyclo)alkyne that participates in the NED interaction. For example, in the case of **8yne** and **9yne**, FMO_**yne**_ refers to LUMO+1_**yne**_ and not LUMO_**yne**_ because the former has the correct symmetry to favorably overlap, and thus, interact with HOMO_**Az**_. The most reactive alkyne, **7yne**, has the smallest NED–FMO energy gap (Δ*ϵ*=8.6 eV) and greatest orbital overlap (*S*=0.15). As the ring size increases from **7yne** to **8yne** and **9yne**, the NED–FMO gaps increase from 8.6 to 9.4 and 9.5 eV, respectively, due to higher lying cycloalkyne virtual orbitals, which result from a smaller bonding admixture between σ* and π* orbitals (see above for details). Also, there is a continuous decrease in orbital overlap upon increasing ring size, due to the shape of the FMO shown in Figure 6. The groups of both Houk and Hoffmann noted that, upon bending acetylene from 180 to 120°, the π and π* orbitals hybridized opposite to the direction of the bending hydrogen atoms.^37^ This hybridization gives rise to π‐HOMO and π‐LUMO lobes that overlap to a greater degree with the respective FMOs on **Az**; an effect also described by Fukui et al.^39^ Hence, cycloadditions involving **8yne** to **9yne** proceed with diminished orbital overlaps of 0.13 and 0.11, respectively, due to smaller alkyne distortion than that of **7yne**. The least reactive alkyne, **2yne**, has the largest HOMO_**Az**_–FMO_**yne**_ gap of 10.3 eV and the least efficient orbital overlap of *S*=0.10.

The inverse electron demand (IED) interaction for these cycloadditions occurs between the HOMO_**yne**_ and LUMO_**Az**_ (Figure 5 b). Again, derivative **7yne** has the smallest IED–FMO energy gap (Δ*ϵ*=6.8 eV) and most favorable overlap (*S*=0.18). The HOMOs of cycloalkynes **8yne** and **9yne** are lower lying than that of **7yne**. This leads to larger, less stabilizing IED–FMO energy gaps of 7.0 and 7.1 eV for **8yne** and **9yne**, respectively. The computed orbital overlaps for **8yne** (*S*=0.16) and **9yne** (*S*=0.15) are also less stabilizing relative to that of **7yne** (Figure 6). Derivatives **2yne** and **9yne**, which have similar orbital interaction curves, also have similar IED–FMO gaps of 7.1 eV and orbital overlaps of *S*=0.15. The enhanced orbital interactions of **7 yne**, compared with those of the other alkynes, are a direct result of the smaller FMO energy gap and better FMO overlap. Both normal and inverse donor–acceptor FMO interactions systematically decrease as the *S* ^2^/Δ*ϵ* term^40^ decreases from **7yne** to **2yne**.

## Conclusion

This study has revealed, for the first time, that the enhanced cycloaddition reactivity of cycloalkynes originates to a substantial, and in some cases predominant, extent from an enhancement of stabilizing orbital interactions, and not just from a reduced activation strain, which has historically been used to rationalize accelerated cycloaddition reactivity. We have arrived at this novel insight on the basis of detailed quantum chemical analyses of the factors that controlled the reactivities of acyclic and cyclic alkynes in 1,3‐dipolar cycloaddition reactions with **Az**. Ring size and, therefore, geometrical distortion, of the cycloalkyne has a profound impact on the cycloaddition reaction rates, which span 16 orders of magnitude between the unstrained, 2‐butyne (**2yn**), and strained, cycloheptyne (**7yne**), derivatives.

Our distortion/interaction–activation strain analyses revealed that the enhanced reactivity of cyclic, relative to acyclic, alkynes arose from three distinct mechanisms, each of which depended on the degree of geometrical predistortion: 1) a reduced activation strain, or distortion energy, because of a reduced need to bend the substituents at the triple bond away from the azide dipole; 2) a smaller HOMO–LUMO gap, mainly by stabilizing the cycloalkyne π‐LUMO; and 3) an enhanced orbital overlap, resulting from the polarization of the π‐HOMO and π‐LUMO lobes on the external π‐face, pointing to the FMOs of the azide dipole.

In summary, our results firmly established that SPAACs benefited from both reduced strain, or distortion energy, and enhanced orbital interactions. We envisage that the identified orbital interactions can be further enhanced by introducing electronically diverse functionalities. The ability to tune the strength of these primary orbital interactions can be a useful tool for the design of bioorthogonal reactions with tailored reaction rates.

## Conflict of interest

The authors declare no conflict of interest.

## Supporting information

As a service to our authors and readers, this journal provides supporting information supplied by the authors. Such materials are peer reviewed and may be re‐organized for online delivery, but are not copy‐edited or typeset. Technical support issues arising from supporting information (other than missing files) should be addressed to the authors.

Supplementary
