# Supplementary material for: Structural Distortion of Cycloalkynes Influences Cycloaddition Rates both by Strain and Interaction Energies
Source: Chemistry. 2019 Mar 27;25(25):6342–8. doi: 10.1002/chem.201900295 (PMC6519225; doi:10.1002/chem.201900295)
Supplement: Supplementary file 1 — Supplementary [file CHEM-25-6342-s001.pdf]

CHEMISTRY
A European Journal

Supporting Information

Structural Distortion of Cycloalkynes Influences Cycloaddition Rates both by Strain and Interaction Energies

Trevor A. Hamlin+,[a] Brian J. Levandowski+,[b] Ayush K. Narsaria,[a] Kendall N. Houk,*[b] and F. Matthias Bickelhaupt*[a, c]

chem_201900295_sm_miscellaneous_information.pdf

Contents

Figure S1. Strain analysis of **Az** upon reacting with alkynes (black, **2yne**; blue, **7yne**; green, **8yne**; red, **9yne**) computed at M06-2X/TZ2P//M06-2X/6-31+G(d).

Table S1. Cartesian coordinates, electronic energies, enthalpies, Gibbs free energies, the number and wavelength of the imaginary vibrational frequencies for all stationary points, computed at M06-2X/6-31+G(d) in the gas phase using Gaussian 09.

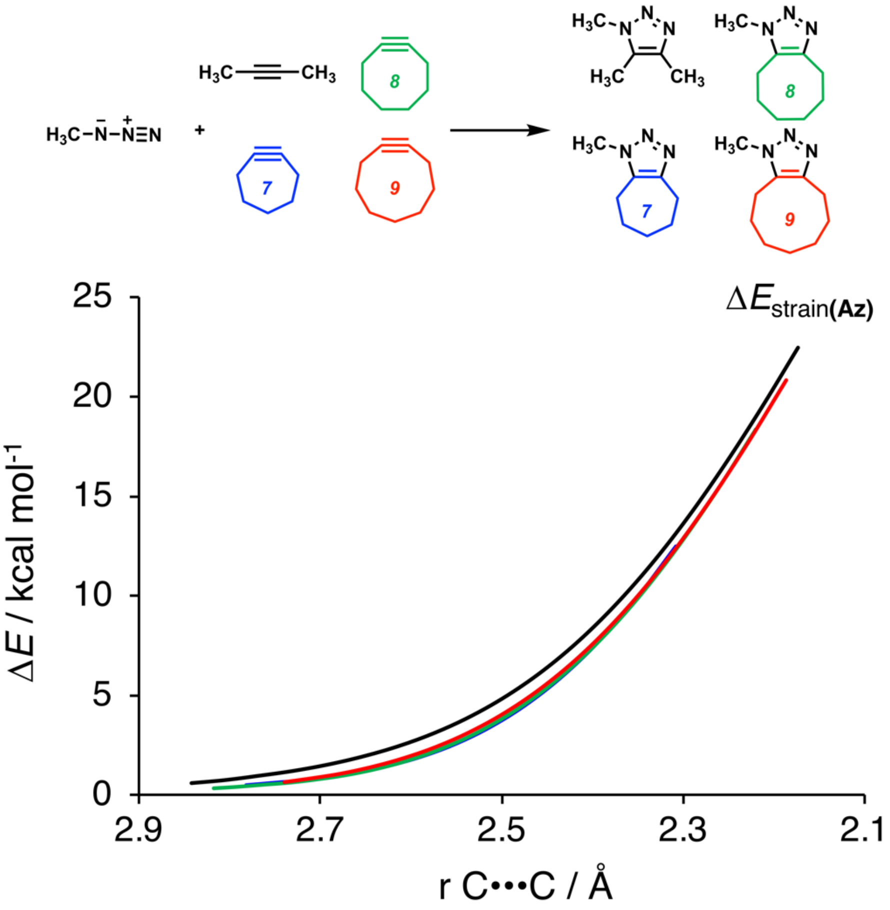

Figure S1. Strain analysis of **Az** upon reacting with alkynes (black, **2yne**; blue, **7yne**; green, **8yne**; red, **9yne**) computed at M06-2X/TZ2P//M06-2X/6-31+G(d).

Table S1. Cartesian coordinates (Å), electronic energies (Hartree), enthalpies (Hartree), Gibbs free energies (Hartree), the number and wavelength of the imaginary vibrational frequencies for all stationary points, computed at M06-2X/6-31+G(d) in the gas phase using Gaussian 09.

Az - azidomethane
E = -203.954015
H = -203.948684
G = -203.980446
$N_{\mathbf{imag}}$ = 0

N	1.22685187	1.32716047	0.00000000
N	2.28652587	1.72437647	-0.00001000
N	0.11921287	0.78815347	-0.00001100
C	-1.00622913	1.73589747	-0.00000700
H	-0.99715913	2.36908947	-0.89421300
H	-1.91402013	1.13516047	0.00000100
H	-0.99715113	2.36909447	0.89419300

2yne - 2-butyne
E = -155.814722
H = -155.808947
G = -155.839613
$N_{\mathbf{imag}}$ = 0

C	-1.52006176	1.62037035	0.00000000
C	-1.52006176	1.62037035	-1.20814200
C	-1.52006176	1.62037035	-2.67335200
C	-1.52006176	1.62037035	1.46521000
H	-0.63478776	1.10925735	-3.06342500
H	-1.52006176	2.64259635	1.85528300
H	-0.63478776	1.10925735	1.85528300
H	-2.40533576	1.10925735	1.85528300
H	-2.40533576	1.10925735	-3.06342500
H	-1.52006176	2.64259635	-3.06342500

7yne - cycloheptyne
E = -272.546783
H = -272.386511
G = -272.423894
$N_{\mathbf{imag}}$ = 0

C	-0.64043211	0.83333332	0.00000000
C	0.58671489	1.64709832	0.05434400
C	0.09773789	3.08694632	-0.25802500
C	-1.23649311	3.52311232	0.41121500
C	-2.57072711	3.08694432	-0.25802200
C	-3.05969711	1.64709332	0.05434400
C	-1.83254711	0.83333532	-0.00001000
H	1.02598689	1.59344832	1.04554400
H	1.34674089	1.33721732	-0.65345400
H	0.87589789	3.78534232	0.04189800
H	-0.00669911	3.18597532	-1.33530900
H	-1.23649211	3.22728432	1.45872900
H	-1.23649511	4.60991232	0.40947700
H	-3.34888811	3.78533632	0.04190800
H	-2.46629411	3.18597832	-1.33530600
H	-3.49896311	1.59343632	1.04554600
H	-3.81972611	1.33721232	-0.65345200

8yne - cyclooctyne
E = -311.856191
H = -311.665467
G = -311.705879
$N_{\mathbf{imag}}$ = 0

C	-1.47376546	2.43827157	0.00000000
C	-0.28600146	2.43826957	-0.07191300
C	1.07045654	2.99084457	-0.16797700
C	0.97214054	4.47563657	0.25126000
C	-0.18518246	5.27841057	-0.39130100
C	-1.57459146	5.27840957	0.31938700
C	-2.73191346	4.47563357	-0.32317400
C	-2.83022446	2.99084057	0.09606200
H	1.77912354	2.46532057	0.46292300
H	1.42911754	2.90905157	-1.18976700
H	0.88449554	4.52609057	1.33319200
H	1.91359654	4.95693557	-0.00316700
H	-0.30195346	4.97402057	-1.42878400
H	0.14711054	6.31211557	-0.42752400
H	-1.45782146	4.97402057	1.35687000
H	-1.90688646	6.31211457	0.35560800
H	-2.64426946	4.52608957	-1.40510500
H	-3.67337046	4.95693057	-0.06874500
H	-3.18888746	2.90904657	1.11785200
H	-3.53888846	2.46531457	-0.53484000

9yne - cyclononyne
E = -351.157172
H = -350.935678
G = -350.978972
$N_{\mathbf{imag}}$ = 0

C	-1.18055558	1.52777776	0.00000000
C	0.00941342	1.52774276	0.00000300
C	0.74035142	4.31066176	0.37143900
C	-0.58560958	4.54694676	-0.40444600
C	-1.91155658	4.31063776	0.37145500
C	-2.88153858	3.31100576	-0.29161200
C	-2.61913558	1.82978476	0.05763500
H	0.53100842	3.98052576	1.38535600
H	1.25963242	5.25939276	0.47265900
H	-0.58562258	5.57067876	-0.76927600
H	-0.58560858	3.92836376	-1.29295400
H	-1.70219458	3.98048276	1.38536200
H	-2.43084658	5.25936176	0.47270300
H	-3.90115858	3.53940876	0.00800600
H	-2.84476458	3.43712576	-1.37026000
H	-2.97416358	1.62565476	1.06386700
H	-3.18093058	1.18391976	-0.60929400
C	1.71033842	3.31103176	-0.29162400
C	1.44797942	1.82981076	0.05765400
H	2.00980742	1.18394976	-0.60925200
H	1.80300042	1.62571576	1.06389600
H	1.67354642	3.43713176	-1.37027400
H	2.72995742	3.53946376	0.00797500

Az-2yne-TS
E = -359.733432
H = -359.722670
G = -359.768193
$N_{\mathbf{imag}}$ = 0, ν = –493.8811i cm^{-1}

C	-0.62500001	-0.46296296	0.00000000
C	-1.60233201	0.29252904	-0.08039000
C	0.78508399	-0.87816996	0.13629900
H	1.09876499	-1.46811996	-0.72979800
H	0.91469799	-1.50695696	1.02214300
C	-2.38567501	1.54057504	-0.13907000
H	-3.08629501	1.60851004	0.70003800
H	-2.96364601	1.59394104	-1.06652800
N	-2.58106801	-2.12769496	-0.21999900
N	-1.47097301	-2.44166996	-0.12358100
N	-3.27145001	-1.09241596	-0.39347800
C	-4.55118501	-1.00961096	0.30344700
H	-4.44164801	-1.14648596	1.38612000
H	-4.94428001	-0.01098796	0.10633800
H	-5.26606301	-1.74022396	-0.08769500
H	-1.72115801	2.40919704	-0.09466800
H	1.44267499	-0.00802196	0.22382400

Az-7yne-TS
E = -476.337540
H = -476.325171
G = -476.375339
$N_{\mathbf{imag}}$ = 0, ν = –381.5793i cm^{-1}

C	-0.67129631	1.41963140	0.01858399
C	-0.36686331	0.23130440	-0.09207601
C	-1.83386031	2.32009940	0.13278399
H	-1.99965231	2.81705740	-0.83195001
H	-1.68415731	3.11315040	0.87317599
C	-0.89196031	-1.13637660	-0.23992301
H	-0.33668531	-1.88190760	0.34141699
H	-0.82698131	-1.43913460	-1.29318301
N	1.95176769	1.45348940	-0.15621101
N	1.43683369	2.47180340	-0.06185001
C	-3.18122931	0.12600340	-0.29913501
C	-3.05314731	1.44618540	0.49996599
H	-3.00004731	1.20229640	1.56935799
H	-3.96527631	2.04022840	0.35881499
H	-4.23807731	-0.16448960	-0.27655001
H	-2.93769631	0.31008140	-1.35583901
C	-2.36787331	-1.08604060	0.21709199
H	-2.39348431	-1.07984960	1.31475099
H	-2.86100531	-2.01245360	-0.10338001
N	1.87570869	0.22425640	-0.36024101
C	2.62799669	-0.62661360	0.56510999
H	3.70661869	-0.52555160	0.41001999
H	2.38232069	-0.40532260	1.61020699
H	2.34108369	-1.65304160	0.33572899

Az-8yne-TS
E = -515.609342
H = -515.595799
G = -515.648746
$N_{\mathbf{imag}}$ = 0, ν = –437.4048i cm^{-1}

C	-1.45833336	-0.03086156	-0.00040400
C	-1.77616636	1.15650344	-0.11914800
C	-0.47230436	-1.11253856	0.17269800
H	-0.77342836	-2.01307256	-0.37331100
H	-0.43727636	-1.39048256	1.23469000
C	-1.49547236	2.59503244	-0.25424500
H	-2.19675336	3.19968944	0.33354100
H	-1.63239536	2.88708444	-1.30401800
N	-4.03446636	-0.16242356	-0.21992000
N	-3.39008536	-1.10768956	-0.07794400
C	1.38819164	0.68641544	0.32287200
C	0.91795164	-0.64684956	-0.28982400
H	1.63658864	-1.43660956	-0.03735300
H	0.91958264	-0.56272856	-1.38432400
H	1.02356164	0.75285244	1.35785000
H	2.48079264	0.64250444	0.39608000
C	1.02133764	1.98850444	-0.44033200
C	-0.05374536	2.89312544	0.19339400
H	0.00271964	2.80883044	1.28628000
H	0.16052564	3.94129644	-0.04948300
H	1.93480264	2.58705944	-0.53170600
H	0.72320564	1.74162144	-1.46880800
N	-3.97594736	1.06679044	-0.45544800
C	-4.90704036	1.92711944	0.27171600
H	-4.81423836	1.80539144	1.35769200
H	-4.65257036	2.95259244	0.00145600
H	-5.94204136	1.73973144	-0.03095200

Az-9yne-TS
E = -554.877499
H = -554.863134
G = -554.917466
$N_{\mathbf{imag}}$ = 0, ν = –452.0438i cm^{-1}

C	-1.30401237	1.31161596	0.01716999
C	-1.03096337	0.10390796	-0.01371101
C	-2.18163037	2.50124196	0.08317299
H	-1.82110237	3.26999296	-0.60886301
H	-2.10769537	2.93597396	1.08909899
C	-1.27622237	-1.35328904	-0.03996201
H	-0.62441637	-1.86281304	0.68039299
H	-1.00933037	-1.74024904	-1.03252801
N	1.26810063	1.34049296	-0.16048801
N	0.62365763	2.29752596	-0.08164701
C	-4.24111337	1.09532596	0.72808399
C	-3.64264637	2.14372196	-0.23253101
H	-4.23647837	3.06499496	-0.20811001
H	-3.68812037	1.77736596	-1.26594201
H	-3.47168537	0.78398596	1.44678599
H	-5.04137537	1.55211996	1.32178299
C	-2.74574937	-1.67762404	0.27832199
H	-2.94786737	-1.36639004	1.31088199
H	-2.86824837	-2.76691804	0.25779899
N	1.15322363	0.10154996	-0.32040001
C	2.10347063	-0.76192104	0.37441299
H	3.11644163	-0.63665904	-0.02076301
H	2.10701263	-0.58559704	1.45674999
H	1.78721563	-1.78741204	0.17850299
C	-4.80431537	-0.14594904	0.02348599
C	-3.74886837	-1.00674004	-0.68298401
H	-4.26223637	-1.77329904	-1.27480601
H	-3.19994037	-0.38467204	-1.40152601
H	-5.33509937	-0.76802504	0.75761699
H	-5.55634337	0.17518296	-0.71043001

Az-2yne-adduct
E = -359.883310
H = -359.873784
G = -359.916729
N_{imag} = 0

C	-0.74969804	1.29801027	0.28339089
C	-1.87494704	2.09730727	0.28477189
C	0.69709496	1.66982027	0.28597489
H	1.22150896	1.17175227	-0.53430211
H	1.17734996	1.36694727	1.22151989
C	-2.08533204	3.57395527	0.28549389
H	-2.61614904	3.90506027	1.18525589
H	-2.66476804	3.89862227	-0.58603411
N	-2.44868504	-0.05140873	0.28647489
N	-1.15527204	-0.00307073	0.28442889
N	-2.90062604	1.20723927	0.28710889
C	-4.32283104	1.47395227	0.28471389
H	-4.60496404	2.04719527	1.17190089
H	-4.60609104	2.02884227	-0.61382911
H	-4.82882204	0.50852227	0.29480489
H	-1.12236104	4.08832527	0.25761189
H	0.82779696	2.74937527	0.17022589

Az-7yne-adduct
E = -476.501826
H = -476.491257
G = -476.536674
N_{imag} = 0

C	-1.11067038	0.64390690	0.27497070
C	-0.66455438	1.94987090	0.31430670
C	-2.49326538	0.10840190	0.46815170
H	-2.74266038	0.09753090	1.53931270
H	-2.48714138	-0.93578810	0.13912870
C	-1.38166338	3.24077490	0.54636370
H	-0.73398138	4.07684690	0.25734270
H	-1.58021338	3.36156090	1.62123770
N	1.02796562	0.55990390	-0.07267730
N	-0.04171738	-0.16316210	0.03426770
C	-3.78538938	2.33479890	0.18847770
C	-3.57499138	0.88972990	-0.28417730
H	-3.34170138	0.88940190	-1.35751930
H	-4.52332738	0.35134290	-0.16853930
H	-4.74018838	2.68866390	-0.21890930
H	-3.89862738	2.34785190	1.28327070
C	-2.70582838	3.34502590	-0.22142830
H	-2.50588638	3.24619790	-1.29669730
H	-3.09980038	4.35638990	-0.06480630
N	0.67024762	1.83906290	0.09479470
C	1.67137462	2.88285490	0.01440770
H	2.63059162	2.38951290	-0.14103730
H	1.46250362	3.55094390	-0.82542430
H	1.69929562	3.45774690	0.94338570

Az-8yne-adduct
E = -515.760495
H = -515.748803
G = -515.796662
N_{imag} = 0

C	-1.26930129	2.26079383	0.48695731
C	-1.80848029	3.53127783	0.38337831
C	0.09675571	1.74926283	0.84212331
H	-0.04024729	0.68092383	1.03771031
H	0.45900371	2.19162483	1.77784831
C	-1.28924129	4.91621483	0.61151731
H	-1.74350229	5.33476183	1.52048331
H	-1.61245629	5.56061483	-0.21890069
N	-3.33192429	1.99858883	-0.15402069
N	-2.24366929	1.36734883	0.14546731
C	1.86875071	3.26504383	-0.33108869
C	1.15415471	1.91263983	-0.26601369
H	1.91916171	1.13955883	-0.12441069
H	0.67701571	1.70303183	-1.23250069
H	2.45461971	3.41167583	0.58829431
H	2.59710271	3.21521783	-1.14999669
C	0.97222571	4.49146583	-0.54462769
C	0.23492471	4.97947583	0.72217031
H	0.55694071	4.40271583	1.59639031
H	0.50805171	6.01837983	0.93614331
H	1.59331271	5.30824683	-0.92879569
H	0.24273371	4.26736483	-1.33595469
N	-3.08692729	3.30513983	-0.01652869
C	-4.12850329	4.27267183	-0.28946169
H	-4.31412529	4.89222383	0.59196931
H	-3.84749829	4.91098983	-1.13171169
H	-5.02744229	3.71002283	-0.54062069

Az-9yne-adduct
E = -555.020596
H = -555.007734
G = -555.058035
N_{imag} = 0

C	-1.22233910	1.46210493	0.38795417
C	-0.66905010	0.19155293	0.41289117
C	-2.54299410	2.02500993	0.83322117
H	-2.32904210	3.07333393	1.06417917
H	-2.85666510	1.56734493	1.77862717
C	-1.16636510	-1.15893507	0.83776117
H	-0.82854210	-1.38083307	1.85893617
H	-0.71677210	-1.92327907	0.18877817
N	0.77676490	1.64952193	-0.45048383
N	-0.29418310	2.30786993	-0.14686483
C	-4.79671310	0.90714393	0.05776117
C	-3.72186010	1.98798393	-0.17994183
H	-4.21797610	2.96234393	-0.12054183
H	-3.32621310	1.91863293	-1.20178483
H	-4.86273910	0.70602293	1.13549017
H	-5.77565110	1.31704993	-0.21839483
C	-2.69272510	-1.26032807	0.75352817
H	-3.12752310	-0.54662107	1.45935317
H	-3.00756210	-2.25316707	1.09518117
N	0.56695290	0.37095493	-0.12038583
C	1.60339290	-0.61346307	-0.35349783
H	2.47947290	-0.07240607	-0.71081383
H	1.84555990	-1.13785307	0.57427917
H	1.28500890	-1.33595407	-1.11023783
C	-4.62155310	-0.40839307	-0.71733983
C	-3.21134810	-0.99458507	-0.66471683
H	-3.17666210	-1.92425107	-1.24694083
H	-2.53424010	-0.29596507	-1.17113883
H	-5.34912810	-1.13959307	-0.33944883
H	-4.87912310	-0.23287107	-1.77003783
